# Effects of Berberine and Hwangryunhaedok-Tang on Oral Bioavailability and Pharmacokinetics of Ciprofloxacin in Rats

**DOI:** 10.1155/2012/673132

**Published:** 2012-10-24

**Authors:** Youn-Hwan Hwang, Won-Kyung Cho, Doorye Jang, Jeong-Ho Ha, Kiyoun Jung, Hyo-In Yun, Jin Yeul Ma

**Affiliations:** ^1^KM-Based Herbal Drug Research Group, Korea Institute of Oriental Medicine, Daejeon 305-811, Republic of Korea; ^2^Laboratory of Veterinary Pharmacology and Toxicology, College of Veterinary Medicine, Chungnam National University, Republic of Korea

## Abstract

Hwangryunhaedok-Tang (HR) and berberine-containing single herbs are used to treat bacterial infection and inflammatory diseases in eastern Asia. The combination of berberine-containing herbal medicines and ciprofloxacin can be an excellent antibacterial chemotherapy against multidrug resistance bacteria. To evaluate the pretreatment effect of berberine and HR, vehicle, berberine (25 and 50 mg/kg/day), and HR (1.4 g/kg/day) were daily administered to rats for five consecutive days. On day 6, ciprofloxacin was administered (10 mg/kg, i.v. and 20 mg/kg, p.o.) to rats. To assess cotreatment effect of berberine and ciprofloxacin, berberine (50 mg/kg) and ciprofloxacin (20 mg/kg) were coadministered by single oral gavage. Pharmacokinetic data were estimated by noncompartmental model. Compared with ciprofloxacin alone (control group), coadministration of berberine (50 mg/kg) and ciprofloxacin significantly decreased *C*_max_ of ciprofloxacin (*P* < 0.05). In addition, the pretreatment of berberine (50 mg/kg/day) and HR (1.4 g/kg/day) significantly decreased *C*_max_ and AUC_0→*∞*_, compared with control group (*P* < 0.05). The oral bioavailability of ciprofloxacin was reduced by cotreatment of berberine and pretreatment of berberine and HR. Our results suggest that the expression of P-glycoprotein and organic anion and/or organic cation transporters (OAT/OCT) could take a role in reduced oral bioavailability of ciprofloxacin by berberine and HR.

## 1. Introduction

Hwangryunhaedok-Tang (HR, Huang-Lian-Jie-Tang in Chinese or Oren-gedoku-to in Japanese), an important multiherb prescription in eastern Asia, is composed of Coptidis Rhizoma, Scutellariae Radix, Phellodendri Cortex, and Gardeniae Fructus [1]. HR has been traditionally used for the treatment of gastrointestinal disorders, cardiovascular disease, inflammatory diseases, and Alzheimer's diseases [2]. Among four single herbs in HR, Coptidis Rhizoma and its constituent berberine, which is a major bioactive cationic isoquinoline alkaloid, have potent broad-spectrum antimicrobial activity against bacteria, fungi, protozoans, helminth, chlamydia, and viruses [3–5]. Especially, berberine alone or in combination with conventional antibiotics including ciprofloxacin significantly increased their antibacterial activity against multidrug-resistant bacteria species [6]. In this point, berberine-containing herbal medicines as well as berberine have attracted the treatment of bacterial infection.

Ciprofloxacin, a synthetic fluorinated 4-quinolone, is mainly used for the treatment of respiratory and urinary tract infections [7]. Recently, ciprofloxacin has been studied for the enforcement of its antibacterial activity via the combination of various plant extracts and their components due to the appearance of multidrug-resistant bacteria [8, 9]. The oral absorption of ciprofloxacin, a substrate of one or more active transporters including P-glycoprotein (P-gp, multidrug-resistant protein, MDR1) and organic anion and/or cationic active transporters (OAT/OCT), can be influenced by coadministration with plant extracts [10]. As a use of nonprescription herbal medicines and supplements is getting popular, there are increasing interest about the potentials of herb-drug interaction.

In recent decades, berberine and HR are known as a substrate and/or inhibitor of P-gp *in vitro* and *in vivo* systems [11, 12]. However, there was no available information about the effects of berberine and HR on the oral bioavailability and pharmacokinetics of ciprofloxacin. To gain a better understanding about the herb-drug or drug-drug interaction among berberine, berberine-containing HR and ciprofloxacin, we investigated whether berberine or HR influences the pharmacokinetics of ciprofloxacin after oral administration in rats.

## 2. Materials and Methods

### 2.1. Chemicals and the Preparation of HR

Ciprofloxacin, ofloxacin (internal standard, IS), and berberine were obtained from Sigma-Aldrich Co. (St. Louis, MO, USA). HPLC-grade water, methanol, and acetonitrile were purchased from J.T. Baker Inc. (Philipsburg, NJ, USA). Other chemicals were purchased from Sigma-Aldrich Co.

All herbs were obtained from Yeongcheon traditional herbal market (Yeongcheon, Republic of Korea). Voucher specimens (number 168 for Coptidis Rhizoma, number 166 for Scutellariae Radix, number 170 for Phellodendri Cortex, and number 144 for Gardeniae Fructus) were deposited in the herbarium of KM-Based Herbal Drug Research Group, Korea Institute of Oriental Medicine (KIOM, Daejeon, Republic of Korea). For preparation of HR, four medicinal herbs (150 g each) were mixed and extracted by heating for 3 h in water (1 : 10 w/v). After lyophilization of the extract, brownish powder (116.5 g) of HR was obtained and stored at 4°C before use. The content of berberine in HR (17.42 ± 0.01 mg/g extract) was quantitated according to previous report [13].

### 2.2. Chromatographic Condition and Preparation of Plasma Samples

Plasma concentration of ciprofloxacin was determined using an HPLC-DAD system (Lachrom Elite, Hitach High-Technologies Corp., Tokyo, Japan). Ciprofloxacin and IS were separated using a ZOBRAX Eclipse plus C_18_ column (4.6 mm × 100 mm, 3.5 *μ*m, Agilent Technologies). The mobile phases were consisted of 0.01% trifluoroacetic acid (TFA) in deionized water (A) and acetonitrile : methanol (1 : 1, B) and programmed by 10-10% (v/v) B at 0.0–3.0 min, 10–50% B at 3.0–10.0 min, and 50-50% B at 10.0–16.0 min. The flow rate was 0.8 mL/min.

Plasma samples (100 *μ*L) were mixed with IS (10 *μ*L, 5.0 *μ*g/mL) and 0.1% TFA in acetonitrile (v/v, 200 *μ*L). The mixture was vortexed for 10 min and then centrifuged at 12,000 rpm for 10 min. Then supernatant was transferred and evaporated to dryness under a stream of N_2_ gas at 40°C. The residue was reconstituted with 0.1% TFA in acetonitrile and centrifuged at 12,000 rpm for 10 min. Supernatant (10 *μ*L) was injected into HPLC system and detected at 280 nm.

### 2.3. Experimental Design and Sample Collection

All experiments were performed after the approval of the Institutional Animal Care and Use Committee of KIOM. Male Sprague-Dawley rats (240–250 g) were obtained from Samtaco (Osan, Republic of Korea). After acclimation under laboratory condition for at least 1 week, rats were randomly divided to 6 groups. Experimental groups are summarized in Table 1. Berberine (5 mg/mL), HR (140 mg/mL), and ciprofloxacin (2 mg/mL) were dissolved and diluted in distilled water. Dosing volume was 10 mL/kg. The rats in Group 4, 5, and 6 were pretreated with berberine or HR for 5 consecutive days prior to ciprofloxacin treatment, whereas those in Group 1, 2, and 3 received distilled water (D.W.) instead of berberine or HR during the same period. On day 6, all animals were administered with a single oral gavage of ciprofloxacin (20 mg/kg) with or without berberine. Blood samples (0.3 mL) were collected in ethylediaminetetraacetic acid tubes via tail vein. Blood samples were collected at 0.08, 0.25, 0.5, 0.75, 1, 1.5, 2, 4, 6, 8, and 12 h after the intravenous (i.v.) administration of ciprofloxacin and at 0.17, 0.33, 0.5, 0.67, 1, 1.5, 2, 4, 6, 8, and 12 h after the oral (p.o.) administration of ciprofloxacin. The samples were centrifuged at 12,000 rpm for 10 min. The plasma samples were stored at −80°C until analysis.

### 2.4. Pharmacokinetic and Statistical Analysis

Using PKSolver program, the noncompartmental pharmacokinetic analysis was conducted in accordance with Hwang et al. [14]. The absolute bioavailability of ciprofloxacin (*F*) after berberine- and HR-treatment was determined as *F* = (AUC_p.o._/AUC_i.v._ × Dose_i.v._/Dose_p.o._) × 100.

Data were expressed as mean ± standard deviation, except for elimination half life, for which harmonic mean and pseudostandard deviation were used. Comparison of data on the pharmacokinetic parameters of ciprofloxacin was performed by the analysis of variance or Student's *t*-test using SPSS version 12.0. Statistical significance of results was considered with a probability level of *P* < 0.05.

## 3. Results and Discussion

In a view of pharmacodynamics and pharmacokinetics, the issue of herb-drug interactions is recently focused by a clinician and practitioner of herbal and conventional medicine. In case of no information on herb-drug interaction, the prescription of conventional and/or herbal medicines could result in the decreases of medicinal effects or the increases of side effects [15, 16]. Usually, herbal medicines and supplements have been self-administered to improve general health problem and to reduce side effects of conventional drugs [17]. Especially, berberine and HR are substrates and inhibitors of cytochrome P450 and active transporters, which take a role in an absorption, metabolism, and elimination of drugs. Currently, berberine is used as a dietary supplement (100–200 mg capsule/day) in the United States, although it is not officially approved by the US Food and Drug Administration [18]. In Chinese medicine, its tablet and capsule (0.2–1.0 g/day) have been used to treat various diseases including type 2 diabetes mellitus [19]. In this context, we examined whether pretreatment or cotreatment of their berberine or HR could influence the pharmacokinetics of ciprofloxacin. In particular, ciprofloxacin and HR unlike berberine can be only used after the prescription of clinician. The coadministration of ciprofloxacin and HR in clinical practices is extremely rare. Thus, coadministration of ciprofloxacin and HR was excluded in this study.

**Figure 1:**
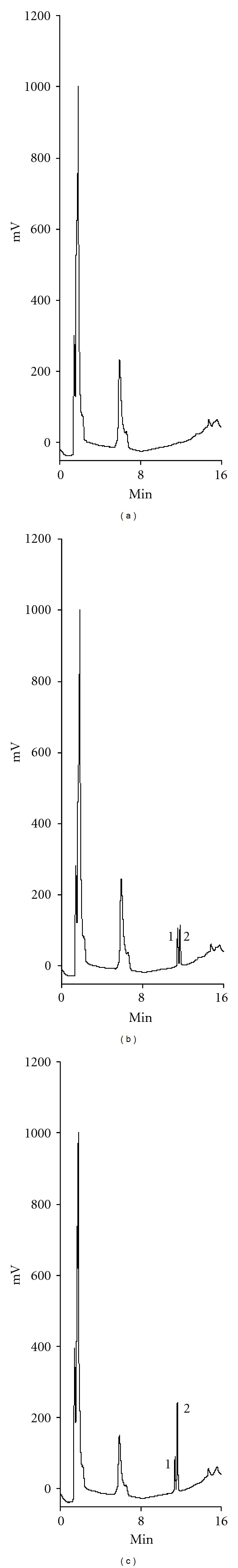
HPLC chromatogram of ciprofloxacin (2) and ofloxacin (IS, 1). (a) Blank plasma; (b) blank plasma spiked with ciprofloxacin and IS; (c) plasma sample of the rat at 1 h after oral administration of ciprofloxacin.

In this study, an HPLC method was validated according to the European Medicines Agency (EMA) guidelines [20]. As shown in Figure 1, ciprofloxacin and IS in rat plasma were detected at 11.3 and 11.7 min, respectively. The linearity of calibration curve (*y* = 2.234*x* + 0.065, *r* < 0.999) was good with a wide range (0.025–5.0 *μ*g/mL). The accuracy and precision of within-run and between-run analysis ranged from −7.94% to 2.51% and from 5.47% to 6.76%, respectively. In between-run analysis, both of the accuracy and precision were less than ±15%. These results imply that the developed and validated method was sufficiently accurate and reproducible in the pharmacokinetic study of ciprofloxacin.

**Figure 2:**
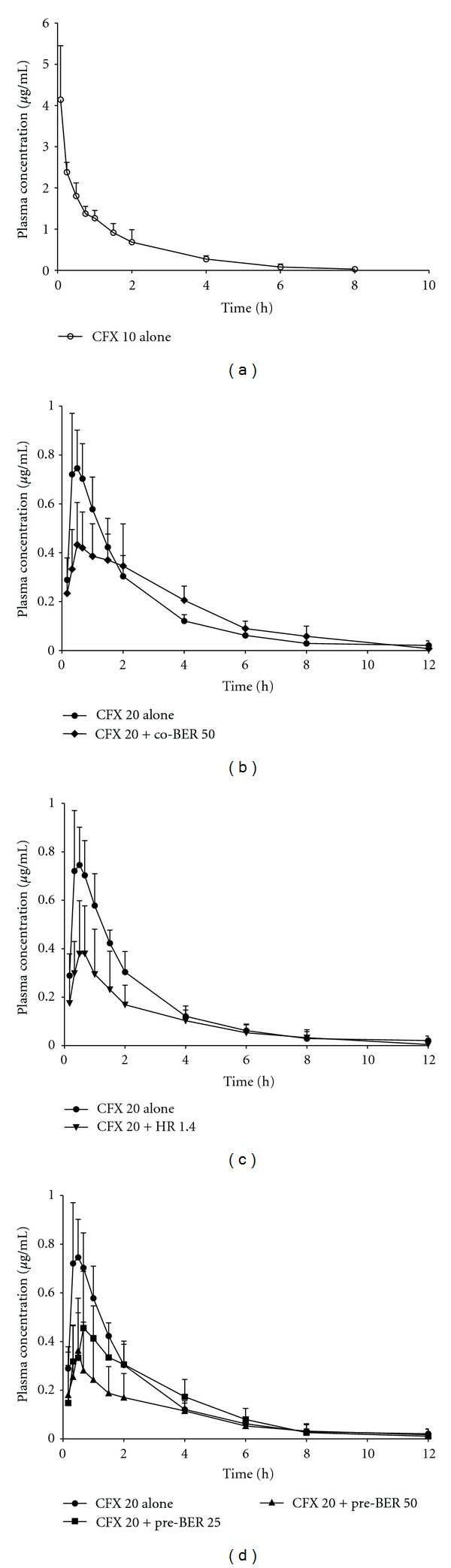
Plasma concentration-time curves of ciprofloxacin after intravenous and oral administration of ciprofloxacin to rats. On day 6, ciprofloxacin was administered after pretreatment of vehicle, berberine, or HR for 5 days. CFX alone and coadministration with berberine received distilled water (D.W.) instead of berberine or HR in the same period. CFX 10 alone (-∘-), CFX (i.v., 10 mg/kg) alone; CFX 20 alone (-●-), CFX (p.o., 20 mg/kg); CFX 20 + Co-BER 50 (-◆-), CFX (p.o., 20 mg/kg) + berberine (50 mg/kg); CFX 20 + Pre-HR (-*▼*-), CFX (p.o., 20 mg/kg) + pretreatment of HR 1.4 (1.4 g/kg/day); CFX 20 + Pre-BER 25 (-■-), CFX (p.o., 20 mg/kg) + pretreatment of berberine (25 mg/kg/day); CFX 20 + Pre-BER 50 (-▲-), CFX (p.o., 20 mg/kg) + pretreatment of berberine (50 mg/kg/day).

Following i.v. administration of ciprofloxacin, the mean plasma concentration-time profile of ciprofloxacin is illustrated in Figure 2(a) and corresponding pharmacokinetic parameters are summarized in Table 2. The initial plasma concentration of ciprofloxacin was 4.14 ± 1.31 *μ*g/mL at 5 min posttreatment and detected up to 8 h. The mean values of *t*_1/2*λ*_*z*__ and AUC_0→*∞*_ were 1.15 h and 4.62 h·*μ*g/mL, respectively. These data are consistent with other reports [21].

To determine the effect of berberine on the intestinal absorption of ciprofloxacin, we coadministered berberine (50 mg/kg) and ciprofloxacin (20 mg/kg) to rat through single oral administration. As shown in Figure 2(b), coadministration of ciprofloxacin and berberine decreased the plasma concentration of ciprofloxacin within 2 h after the administration of ciprofloxacin, comparing with ciprofloxacin alone (control group). Compared with control group, the coadministration of berberine resulted in a decrease of *C*_max⁡_ (*P* < 0.05). However, there were no significant differences in other pharmacokinetic parameters and absolute bioavailability. Ciprofloxacin displays concentration-dependent bacterial killing [22]. In addition, *C*_max⁡_/MIC (minimum inhibitory concentration) index is an important predictor of ciprofloxacin efficacy against ciprofloxacin-susceptible or -resistant pathogens [23]. Szałek et al. [24] reported that the decreased *C*_max⁡_/MIC index of ciprofloxacin may need to verify the assumed administration scheme in patients with cystic fibrosis. These data suggest that the reduced *C*_max⁡_ of ciprofloxacin could lead to the decrease of therapeutic efficacy.

Following pretreatment of berberine or HR during 5 consecutive days prior to the administration of ciprofloxacin, the mean plasma concentration-time curves of ciprofloxacin in each group were obtained, as shown in Figures 2(c) and 2(d). Both berberine and HR obviously decreased plasma concentration of ciprofloxacin, comparing with those of control group. Berberine decreased plasma concentration of ciprofloxacin in a dose-dependent manner. The corresponding pharmacokinetic parameters are summarized in Table 1. The pretreatment of berberine (50 mg/kg/day) or HR significantly decreased the values of *C*_max⁡_ and AUC_0→*∞*_, compared with control group (*P* < 0.05). Moreover, the absolute bioavailability (*F*) of ciprofloxacin was reduced about 40% by the pretreatment of berberine and HR. These findings indicate that the pretreatment of berberine as well as HR can affect the intestinal absorption of ciprofloxacin.

The intestinal absorption of ciprofloxacin involves passive diffusion and P-gp and OAT/OCT efflux pumps in relation with its zwitterionic property. The active transporters take a part in the intestinal elimination of ciprofloxacin in passing the intestinal barrier. P-gp plays an important role for the intestinal secretion of ciprofloxacin and the improvement of antibacterial efficacy [10, 25]. Ciprofloxacin is a substrate of P-gp [26]. Additionally, OAT/OCT pump apart from P-gp mediates the intestinal elimination of ciprofloxacin, based on a mechanism sensitive to quinidine and verapamil [27, 28]. Likewise, berberine is a substrate of P-gp in various species including humans [29–31]. Berberine as a P-gp substrate improved antibacterial efficacy of various antibiotics against MDR1-overexpressing resistant bacteria [9, 32, 33]. Coadministration of berberine dose dependently increased the bioavailability of digoxin and cyclosporin A via the inhibition of intestinal P-gp [11]. Diversely, berberine upregulated the expression of MDR1 in various mammalian cell lines [30, 31, 34]. Moreover, the repeated oral administration of berberine reduced the intestinal absorption of P-gp substrates and increased the expression of MRP in digestive track cancer cells [35, 36]. As shown in Table 2, both coadministration and pretreatment of berberine (25 mg/kg/day and 50 mg/kg/day) significantly decreased the value of *C*_max⁡_ and AUC_0→*∞*_ of ciprofloxacin with the reduction (about 40%) of oral bioavailability (*P* < 0.05). In this respect, the bioavailability of ciprofloxacin decreased by berberine could be not only competitive inhibition as a P-gp and OAT/OCT substrate but also upregulation of P-gp expression.

Since about 40–60% of the dose is excreted via urine, nonrenal clearance of ciprofloxacin is important in total body elimination of ciprofloxacin [37]. Ketoconazole and itraconazole (both are CYP 3A4 inhibitors) significantly decreased total body clearance of ciprofloxacin [7]. Repeated administration of berberine markedly decreased CYP2D6, 2C9, and CYP3A4 activities in human study [38]. On the contrary, berberine did not significantly change in CYP3A activity on the pharmacokinetics of carbamazepine (CYP3A substrate) [11]. Berberine is poorly absorbed into gastrointestinal tract [39, 40] and rapidly excreted through demethylation and glucuronidation in liver [41]. Therefore, further study is needed to clarify whether berberine affects CYP-related metabolism and elimination of ciprofloxacin.

Many researchers have demonstrated herb-drug or drug-drug interaction of HR and single herbs in HR as well as major active compounds. HR increased the bioavailability of verapamil via inhibiting first-pass verapamil metabolism in the intestine [42]. The coadministration of *Scutellaria baicalensis* with cyclosporine decreased the *C*_max⁡_ and AUC of cyclosporine, whereas the coadministration of baicalin and baicalein increased the intestinal absorption of cyclosporine [43]. Baicalin as a substrate of OAT1B1 reduced plasma concentrations of rosuvastatin [44]. Baicalein enhanced the oral bioavailability of tamoxifen on the basis of CYP3A4-medicated metabolism of tamoxifen and P-gp efflux pump [45]. As shown in Table 2, HR significantly decreased the oral bioavailability of ciprofloxacin. Although HR (equivalent to 25 mg/kg/day of berberine) was administered prior to the pharmacokinetic study of ciprofloxacin, *C*_max⁡_, AUC_0→*∞*_, and bioavailability of HR pretreatment were lower than those of control group. Therefore, these results imply that other active compounds, contained in HR, separate from berberine could influence the intestinal absorption and bioavailability of ciprofloxacin via P-gp and OAT/OCT.

## 4. Conclusion

In this study, we first evaluated the effects of berberine and HR on the pharmacokinetics of ciprofloxacin after oral administration. Here, the pretreatment and coadministration of both berberine and HR lowered the AUC and oral bioavailability of ciprofloxacin though their potential for combination chemotherapy. The reduced oral bioavailability of ciprofloxacin by herb-drug or drug-drug interaction may be important for critical care setting to prevent therapeutic failures. Further investigations are required to determine the effects of berberine on the expression of active transporters responsible for intestinal drug absorption.

## Figures and Tables

**Table 1:** Summary of the experimental groups.

Groups	*n*	Treatment^a^
Group 1	4	CFX (i.v., 10 mg/kg)
Group 2	5	CFX (p.o., 20 mg/kg)
Group 3	5	CFX (p.o., 20 mg/kg) + berberine (p.o., 50 mg/kg)
Group 4	5	CFX (p.o., 20 mg/kg) + pretreatment of HR (p.o., 1.4 g/kg/day)
Group 5	5	CFX (p.o., 20 mg/kg) + pretreatment of berberine (p.o., 25 mg/kg/day)
Group 6	5	CFX (p.o., 20 mg/kg) + pretreatment of berberine (p.o., 50 mg/kg/day)

^a^Group 1, 2, and 3 received distilled water (D.W.) instead of berberine or HR in the same period. After 5 consecutive pretreatment of vehicle, berberine, or HR, ciprofloxacin was administered with or without berberine on day 6.

**Table 2:** Pharmacokinetics parameters of ciprofloxacin after intravenous (i.v., 10 mg/kg) and oral (p.o., 20 mg/kg) administration of ciprofloxacin with or without co-treatment and pre-treatment of berberine and HR.

Parameters^a^	i.v.	p.o.				
	—	—	Cotreatment	Pretreatment		
	CFX alone	CFX alone	Berberine (50 mg/kg)	HR (1.4 g/kg/day)	Berberine (25 mg/kg/day)	Berberine (50 mg/kg/day)
*T*_max⁡_ (h)	—	0.60 ± 0.25	0.54 ± 0.16	0.73 ± 0.45	0.70 ± 0.18	0.57 ± 0.09
*C*_max⁡_ (*μ*g/mL)	—	0.84 ± 0.17	0.49 ± 0.19*	0.42 ± 0.23*	0.49 ± 0.21*	0.37 ± 0.22*
*λ*_*z*_ (1/h)	0.61 ± 0.08	0.45 ± 0.18	0.48 ± 0.22	0.45 ± 0.18	0.45 ± 0.11	0.40 ± 0.19
*t*_1/2 *λ*_*z*__ (h)	1.15 ± 0.17	1.55 ± 0.52	1.45 ± 0.46	1.53 ± 0.23	1.54 ± 0.50	1.75 ± 0.37
AUC_0→*∞*_ (h·*μ*g/mL)	4.62 ± 1.06	1.84 ± 0.52	1.87 ± 0.39	1.11 ± 0.56*	1.59 ± 0.42	1.1 ± 0.41*
*F* (%)	—	19.87	20.29	12.00	17.16	11.95

^a^ *T*_max⁡_: time to reach *C*_max⁡_; *C*_max⁡_: maximum plasma drug concentration; *λ*_*z*_: elimination rate constant; t_1/2 λ_z__: elimination half life; AUC_0→∞_: area under the plasma concentration-time curves from time zero to infinity; *F*: absolute bioavailability. *P < 0.05, ciprofloxacin alone versus co- or pre-treatment of berberine and HR. The rats treated with CFX alone and the co-treatment of berberine and ciprofloxacin received vehicle (D.W.) during the same period of pre-treatment groups.
